# Proton Beam Therapy Provides Longer Survival and Preserves Muscle Mass in Hepatocellular Carcinoma Compared to TACE+RFA

**DOI:** 10.3390/cancers17172849

**Published:** 2025-08-30

**Authors:** Takuto Nosaka, Ryotaro Sugata, Yosuke Murata, Yu Akazawa, Tomoko Tanaka, Kazuto Takahashi, Tatsushi Naito, Masahiro Ohtani, Kenji Takata, Tetsuya Tsujikawa, Yoshitaka Sato, Yoshikazu Maeda, Hiroyasu Tamamura, Yasunari Nakamoto

**Affiliations:** 1 Second Department of Internal Medicine, Faculty of Medical Sciences, University of Fukui, Fukui 910-1193, Japan; r-sugata@u-fukui.ac.jp (R.S.); yosukem@u-fukui.ac.jp (Y.M.); aka0124@u-fukui.ac.jp (Y.A.); kawakami@u-fukui.ac.jp (T.T.); tkazuto@u-fukui.ac.jp (K.T.); naitot@u-fukui.ac.jp (T.N.); mohtani@u-fukui.ac.jp (M.O.); 2 Department of Radiology, Faculty of Medical Sciences, University of Fukui, Fukui 910-1193, Japan; tkenji@u-fukui.ac.jp (K.T.); awaji@u-fukui.ac.jp (T.T.); 3 Proton Therapy Center, Fukui Prefectural Hospital, Fukui 910-8526, Japan; y-satou-xn@pref.fukui.lg.jp (Y.S.); y-maeda-ce@pref.fukui.lg.jp (Y.M.); h-tamamura-8e@pref.fukui.lg.jp (H.T.); 4 Division of Human and Artificial Intelligent Systems, Graduate School of Engineering, University of Fukui, Fukui 910-8507, Japan

**Keywords:** hepatocellular carcinoma, proton beam therapy, transcatheter arterial chemoembolization, radiofrequency ablation, psoas muscle

## Abstract

Background: Proton beam therapy (PBT) provides excellent tumor control with minimal hepatic toxicity in patients with unresectable hepatocellular carcinoma (HCC), by minimizing radiation exposure to non-cancerous liver tissue. Progressive skeletal muscle loss, often seen in cirrhosis and HCC, can negatively impact treatment outcomes and survival. This study compared the efficacy and safety of PBT with transarterial chemoembolization (TACE) combined with radiofrequency ablation (RFA) in patients with unresectable HCC. Methods: A total of 91 patients (PBT/TACE+RFA, *n* = 41/50) ineligible for surgery or RFA alone were retrospectively analyzed, with propensity score matching applied to adjust for differences in baseline characteristics, resulting in matched groups of 33 patients each. The cross-sectional area of the psoas muscle at the third lumbar vertebra was assessed using computed tomography. Results: PBT resulted in longer overall survival (OS) and fewer hepatic and systemic adverse events compared to TACE+RFA, with no grade 3 or higher toxicities observed in the PBT group. Importantly, psoas muscle size remained stable after PBT, even in patients with tumors ≥ 3 cm, whereas TACE+RFA led to significant muscle loss regardless of tumor size, which was associated with poorer prognosis. These findings suggest that, for patients with unresectable HCC not adequately controlled by RFA alone, PBT may improve OS and help preserve muscle mass, while offering lower toxicity and more favorable clinical outcomes than TACE+RFA. Conclusions: Overall, PBT may represent an effective strategy for managing unresectable HCC.

## Simple Summary

Proton beam therapy (PBT) is known to achieve excellent tumor control with minimal damage to non-cancerous liver tissue in patients with hepatocellular carcinoma (HCC) who are not eligible for surgery. Loss of skeletal muscle can worsen treatment outcomes and survival in patients with chronic liver disease and HCC. This study compared PBT with a combination of transarterial chemoembolization (TACE) and radiofrequency ablation (RFA) in patients with unresectable HCC who could not be treated with surgery or RFA alone. The results showed that PBT led to better survival and preserved psoas muscle size. Psoas muscle size remained stable after PBT, whereas TACE+RFA caused a reduction in psoas muscle size, linked to poorer outcomes. Hepatic and systemic side effects were less frequent with PBT. These findings suggest that PBT may be a promising treatment for certain patients with unresectable HCC, offering effective tumor control and preservation of psoas muscle size.

## 1. Introduction

Liver cancer is the sixth most prevalent neoplasm and the third most common cause of cancer-related mortality. Hepatocellular carcinoma (HCC) accounts for approximately 85–90% of liver cancer cases [1]. Progressive and generalized loss of skeletal muscle mass is a serious life-threatening complication of cirrhosis and HCC [2,3]. Decreased skeletal muscle mass increases the risk of adverse outcomes, such as falls, fractures, infections, hepatic encephalopathy, and decreased liver function. It also negatively impacts clinical outcomes, including treatment efficacy and survival [4,5,6]. The annual rate of change in skeletal muscle area is an important factor in estimating the risk of major outcomes in patients with HCC and lung cancer [2,7,8].

In recent years, proton beam therapy (PBT) has been utilized for HCC treatment, with several studies reporting its local control effects and safety [9,10]. Among radiation therapies, PBT achieves excellent long-term tumor control with minimal toxicity in patients with unresectable HCC [11]. PBT reduces the risk of radiation-induced liver disease (RILD) because the radiation dose around normal tissue is low [12]. This is particularly important in the treatment of patients with HCC who have chronic liver disease. We previously reported that PBT is effective for local control without reducing hepatic reserve function after treatment [13]. However, the effect of PBT on skeletal muscle volume in patients with HCC remains unknown.

Radiofrequency ablation (RFA) is the most commonly used thermal ablation therapy for the local treatment of HCC. However, in the case of RFA monotherapy, the local control rate is low for large lesions (≥3 cm) [14] and small lesions (≤3 cm) that are adjacent to large blood vessels, such as the portal vein [15], or are located below the diaphragm [16]. To address these issues, combined treatment using transarterial chemoembolization (TACE) and RFA is being administered. This combination is based on the theory that TACE reduces the cooling effect of hepatic blood flow from the hepatic artery and enhances the necrotic effect of RFA therapy [17,18]. TACE+RFA significantly improve overall survival (OS) and local tumor control compared with TACE alone in patients with HCC at Barcelona Clinic Liver Cancer (BCLC) stage B [18,19]. However, TACE injures surrounding liver parenchymal cells, worsens hepatic functional reserve, and results in adverse events, which may worsen the prognosis for patients with HCC who have chronic liver disease [20]. In the TACE+RFA group of the present study, all patients underwent RFA following TACE as part of a preplanned combination therapeutic strategy. Although ablation monotherapy is generally recommended as the first-line treatment for patients with BCLC stage 0 or A, its efficacy may be compromised in specific anatomical contexts—such as large lesions, tumors adjacent to major vessels like the portal vein, and those located in subdiaphragmatic regions. Therefore, combination therapy with TACE and RFA has been increasingly adopted, even in early-stage HCC. Indeed, several clinical studies have reported that TACE combined with RFA confers superior progression-free and overall survival compared to TACE alone, even in early-stage disease [18,21,22]. Accordingly, in this study, TACE was deliberately administered prior to RFA to maximize local tumor control.

This study aimed to compare treatment efficacy and long-term prognosis of PBT and TACE+RFA therapy for unresectable HCC that was uncontrolled by RFA alone. Additionally, to understand the influence of sarcopenia in the prognosis of the treated patients, the study examined the association of changes in the psoas muscle area and survival time.

## 2. Patients and Methods

### 2.1. Patients

Between January 2010 and June 2024, 43 patients with HCC treated with PBT and 53 patients who underwent TACE followed by RFA (TACE+RFA) were included in this analysis. The criteria for inclusion in the PBT and TACE+RFA groups were as follows: (1) patients ineligible for surgical resection due to comorbidities, such as cardiopulmonary impairment, or those who declined surgery; (2) tumors located near major blood vessels, such as the portal vein, or in subphrenic regions; (3) an Eastern Cooperative Oncology Group (ECOG) performance status of 0–2; (4) absence of uncontrolled ascites; (5) no extrahepatic spread of the disease; (6) no prior local recurrence at the same site following resection or RFA; and (7) liver function categorized as Child-Pugh class A or B. Exclusion criteria were as follows: patients for whom computed tomography (CT) images of the psoas muscle were not available both before treatment and approximately one year after treatment. Based on this criterion, 2 patients were excluded from the PBT group and 3 from the TACE+RFA group. As a result, a total of 41 patients in the PBT group and 50 patients in the TACE+RFA group were included in the final analysis (Supplementary Figure S1). Patient demographics and clinical data are summarized in Table 1. HCC diagnosis was confirmed based on typical imaging findings from CT or magnetic resonance imaging (MRI), in accordance with the management guidelines provided by the American Association for the Study of Liver Diseases (AASLD) [23]. In cases where imaging findings were atypical, a percutaneous needle biopsy of the liver tumor was performed to obtain histopathological confirmation of HCC. This retrospective study was conducted following approval from the Research Ethics Committee of the University of Fukui (approval number: 20220071) and the Ethics Committee of Fukui Prefecture Hospital (approval number: 22-21). The study information was made publicly available at http://research.hosp.u-fukui.ac.jp/wp-content/uploads/2022/08/20220071.pdf (accessed on 9 August 2022). For treatment selection between the PBT group and the TACE+RFA group, (1) PBT was selected when tumors were located in areas where RFA was difficult (near the portal vein or just below the diaphragm); (2) TACE+RFA was selected when tumors were located near risk organs such as the gastrointestinal tract, lungs, or heart, making PBT irradiation difficult. These decisions were made through multidisciplinary conferences and with the patient’s consent.

### 2.2. Assessment of Hepatic Reserve Function

The albumin–bilirubin (ALBI) score was calculated using the following formula: ALBI score = (log10 bilirubin (µmol/L) × 0.66) + (albumin (g/L) × −0.085). Based on the score, ALBI grades were assigned as follows: ≤−2.60, Grade 1; >−2.60 to ≤−1.39, Grade 2; and >−1.39, Grade 3 [24]. Grade 2 was further subdivided into two subcategories, 2a and 2b, using a previously established ALBI score cutoff value of −2.270. These four categories were collectively referred to as modified ALBI (mALBI) grades [25].

### 2.3. Proton Beam Therapy

The patient setup, planning images, and PBT procedures have been previously detailed [13,26]. Planning used respiratory-synchronized 4D-CT (Aquilion LB TSX-201A: Canon Medical Systems Co., Tochigi, Japan) combined with a breathing synchronization method that monitored abdominal skin surface motion using a laser sensor (AZ-733V: Anzai Medical Co., Tokyo, Japan). The CT scan was reconstructed during the expiration phase when respiratory motion was minimal, and the target volumes were contoured. The planning target volume (PTV) was defined by a 0.5 cm margin around the internal target volume. Proton treatment planning was performed for the passive scattering method using the XiO^®^-*n* system (Elekta Corp., Stockholm, Sweden), with dose calculations based on the pencil beam algorithm. The prescribed dose was set to the geometrical center of the PTV, using several beam angles. The number of beam angles was 2 or 3. The proton dose distribution was formed with the patient’s collimator or multi-leaf collimators and patient’s bolus. A field margin of collimators was adjusted to achieve at least 97.5% volume coverage (V95%) of the PTV. A range margin of 2−3% along the beam direction, and a smearing value of 6−12 mm for the bolus were set so as to maintain the dose coverage of the target volume against daily movement of the target throughout the entire treatment [26]. The details of dose distribution shaping, coverage optimization, and beam arrangement have been described elsewhere [13,26]. In brief, planning aimed to secure adequate PTV coverage while minimizing exposure to the normal liver and gastrointestinal (GI) tract. Proton beams were delivered with a respiratory-gated system (Hitachi Corporation, Tokyo, Japan). Respiratory-gated PBT protocols, ranging from 66.0 to 80.5 CGE over 10–38 fractions depending on tumor location, have been reported previously [27]. Fractionation schedules were selected according to tumor proximity to critical organs, with modifications applied as necessary to reduce the risk to surrounding tissues or to accommodate patient condition.

### 2.4. Transarterial Chemoembolization

TACE was performed according to the protocols described in our previous study [13]. The femoral artery was accessed percutaneously, and diagnostic angiography was used to delineate tumor-supplying vessels. The technical specifics for conventional TACE (cTACE)—selective catheterization with a miriplatin–lipiodol emulsion followed by gelatin sponge particle embolization—and for drug-eluting bead TACE (DEB-TACE) with epirubicin-loaded beads have been detailed previously [13]. Microcatheters were navigated over a guidewire into the tumor feeders, and drug-eluting beads were delivered until angiographic tumor staining resolved. When vascular lakes were observed, additional embolization with gelatin sponge particles (Gelpart^®^) was performed.

### 2.5. Radiofrequency Ablation

RFA was performed according to the protocols described in our previous study [13]. After TACE for HCC, RFA was conducted on the same lesion once treatment response and the patient’s condition were confirmed to be stable. The procedural details of percutaneous RFA using a cool-tip electrode under ultrasound guidance, including device specifications, ablation settings, and post-procedural assessment with contrast-enhanced CT, have been reported previously [13]. In brief, electrode type and energy output were selected according to tumor size and location to ensure an adequate ablation margin, and repeat ablation was performed during the same hospitalization if margins were insufficient.

### 2.6. Propensity Score Matching

To minimize selection bias, propensity score matching (PSM) was applied to compare the PBT group and the TACE+RFA group (Table 1 and Table 2). The propensity scores for treatment options were estimated using multiple logistic regression analysis. The propensity scores were calculated using a propensity model that included age, gender, ECOG-PS, etiology, muscle atrophy, AFP, ALBI score, tumor size, number of treated lesions, and vascular invasion. The match was made using a 1:1 matching scheme, and the caliper width was equal to 0.2 of the logit standard deviation of the propensity score. In the matched sample, the absolute standardized differences in the mean values and proportions of these variables were all less than 0.10, indicating balanced groups.

### 2.7. Etiology of Liver Diseases

The etiology of HCC was classified as hepatitis C virus (HCV) for patients testing positive for anti-HCV antibodies (HCV Ab) and as hepatitis B virus (HBV) for those testing positive for hepatitis B surface antigen (HBsAg). Patients who tested negative for both anti-HCV Ab and HBsAg were categorized as non-B, non-C (NBNC).

### 2.8. Measurement of Psoas Muscle Area

**Figure 1:**
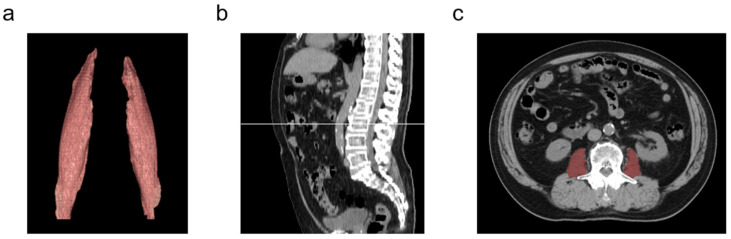
Evaluation of the psoas muscle area in a representative patient. (**a**) Image of the psoas muscle isolated using an automatic analysis program on the Ziostation2 workstation. (**b**) Sagittal computed tomography (CT) image; the white line indicates the mid-level of the third lumbar vertebra (L3). (**c**) Axial CT image with the psoas muscle highlighted in red.

The area of the psoas muscle was measured from CT images using Ziostation2 (Ziosoft, Tokyo, Japan). Using the automatic analysis program on the Ziostation2 workstation, the psoas muscle was isolated, and the cross-sectional area at the mid-level of the third lumbar vertebra (L3) was measured (Figure 1). Psoas muscle area change was measured by calculating the percentage change from baseline to post-treatment in the cross-sectional area of the psoas muscle at the level of the L3 on CT images obtained before and after treatment. CT images obtained between 8 and 14 months after treatment were analyzed, with this range reflecting the variability in follow-up timing among patients. The median follow-up period was approximately 12 months; therefore, this time point is referred to as “approximately 1 year” throughout the manuscript for consistency. The duration of CT follow-up was confirmed for each patient, and the median and interquartile range of the follow-up period were calculated. A comparison of the duration of CT follow-up between the PBT group and the TACE+RFA group before and after PSM showed no significant difference (Table 1). The measured psoas muscle area (cm^2^) was divided by the square of the patient’s height (m) to calculate the psoas muscle index (PMI). Muscle atrophy diagnosis was based on the diagnostic criteria for sarcopenia in patients with liver disease established by the Japan Society of Hepatology, with cutoff values of 6.36 cm^2^/m^2^ for men and 3.92 cm^2^/m^2^ for women [28].

### 2.9. Evaluation of Outcomes

Local progression-free survival (PFS) was described as the period beginning at the start of PBT or TACE and ending at the earliest event of local disease progression or death from any cause. Local disease progression was identified either by the appearance of new tumors or by the enlargement of existing lesions within the treatment field of PBT or TACE+RFA. OS was measured from the initiation of PBT or TACE to the date of death from any cause or the last recorded visit. Patients lost to follow-up were censored at their last known survival date, and those still alive were censored at the data cut-off point.

### 2.10. Evaluation of Adverse Events

Adverse events were assessed according to the Common Terminology Criteria for Adverse Events (CTCAE), version 5.0. ALT or AST elevation was defined as exceeding the upper limit of normal (ULN) and classified as: Grade 1 (ULN–3 × ULN), Grade 2 (3–5 × ULN), Grade 3 (5–20 × ULN), and Grade 4 (>20 × ULN). Albumin decrease was graded as: Grade 1 (from lower limit of normal [LLN] to 3.0 g/dL), Grade 2 (2.0–3.0 g/dL), and Grade 3 (<2.0 g/dL). Bilirubin elevation was graded as: Grade 1 (>ULN–1.5 × ULN), Grade 2 (1.5–3.0 × ULN), Grade 3 (3.0–10.0 × ULN), and Grade 4 (>10.0 × ULN). These laboratory values were evaluated within four weeks after initiation of treatment.

### 2.11. Statistical Analyses

All statistical analyses were performed with GraphPad Prism software, version 10.4.1 (GraphPad Software, San Diego, CA, USA). Comparisons of categorical variables were carried out using the Mann–Whitney U test, Fisher’s exact test, chi-square test, and the log-rank test. Survival curves were estimated with the Kaplan–Meier method. Statistical significance was set at *p* < 0.05. A propensity score analysis was performed using the Easy R version 4.3.1 software (Jichi Medical University Saitama Medical Center, Saitama, Japan) [29]. Clinical factors of survival were identified using the Cox proportional hazards model. Statistical analyses were performed using Prism software.

## 3. Results

### 3.1. Therapeutic Effects of PBT and TACE+RFA

**Figure 2:**
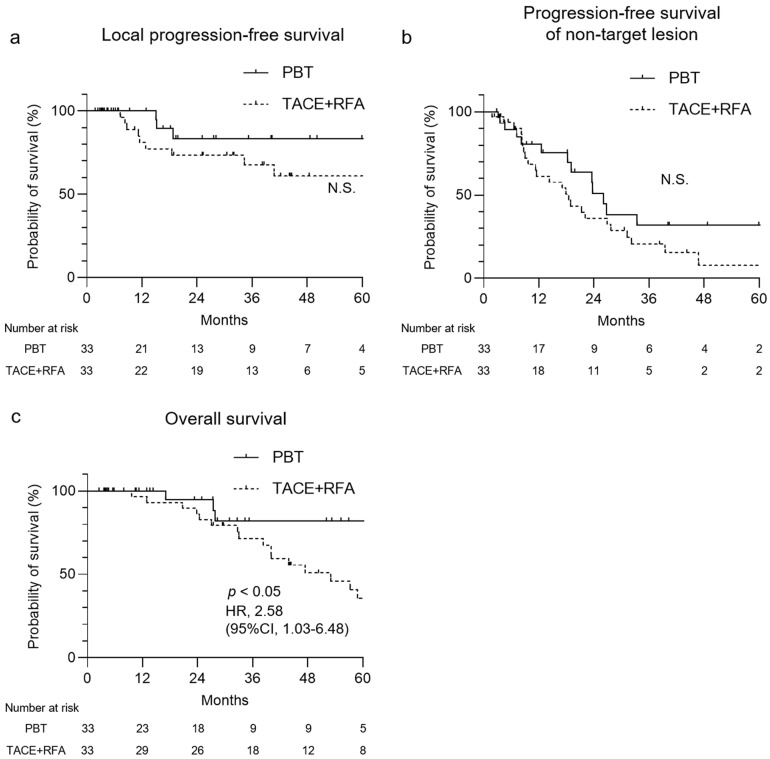
Progression-free survival (PFS) and overall survival (OS) for patients with HCC treated with PBT and TACE+RFA. (**a**,**b**) Kaplan–Meier curves of PFS of target lesions (**a**) and non-target lesions (**b**) for patients with HCC treated with PBT and TACE+RFA. (**c**) Kaplan–Meier curves of OS for patients with HCC treated with PBT and TACE+RFA. CI, confidence interval; HR, hazard ratio; PBT, proton beam therapy; TACE, transarterial chemoembolization; RFA, radiofrequency ablation; N.S., not significant.

To reduce selection bias between the PBT group and the TACE+RFA group, PSM was performed (Table 1 and Table 2). The detailed methodology of PSM is described in the Materials and Methods section. There was no significant difference between the PBT and TACE+RFA groups in any variable including age, gender, ECOG-PS, etiology, muscle atrophy, AFP, ALBI score, tumor size, number of treated lesions, and vascular invasion. The clinical course of patients with HCC after PBT and TACE+RFA was investigated. In both treatments, >61% of the treated tumor was controlled at 60 months, and no difference in local PFS was observed (Figure 2a). The PFS for non-target lesions did not differ between the two treatments (Figure 2b). Among patients who received PBT, the 60-month post-treatment OS rate was 82% (Figure 2c). Contrastingly, the 60-month post-treatment OS rate for patients who received TACE+RFA was 35%. We performed a Cox regression analysis including the following variables: age, gender, ECOG performance status, etiology, muscle atrophy, AFP level, modified ALBI grade, tumor size, number of treated lesions, vascular invasion, and treatment modality (Table 3). This analysis demonstrated that PBT and mALBI grade 1/2a were independent prognostic factors significantly associated with overall survival. In summary, while the recurrence rate of target lesions was comparable between the groups of patients with HCC, the PBT group demonstrated superior overall survival.

### 3.2. Changes in Psoas Muscle Size After Treatments

**Figure 3:**
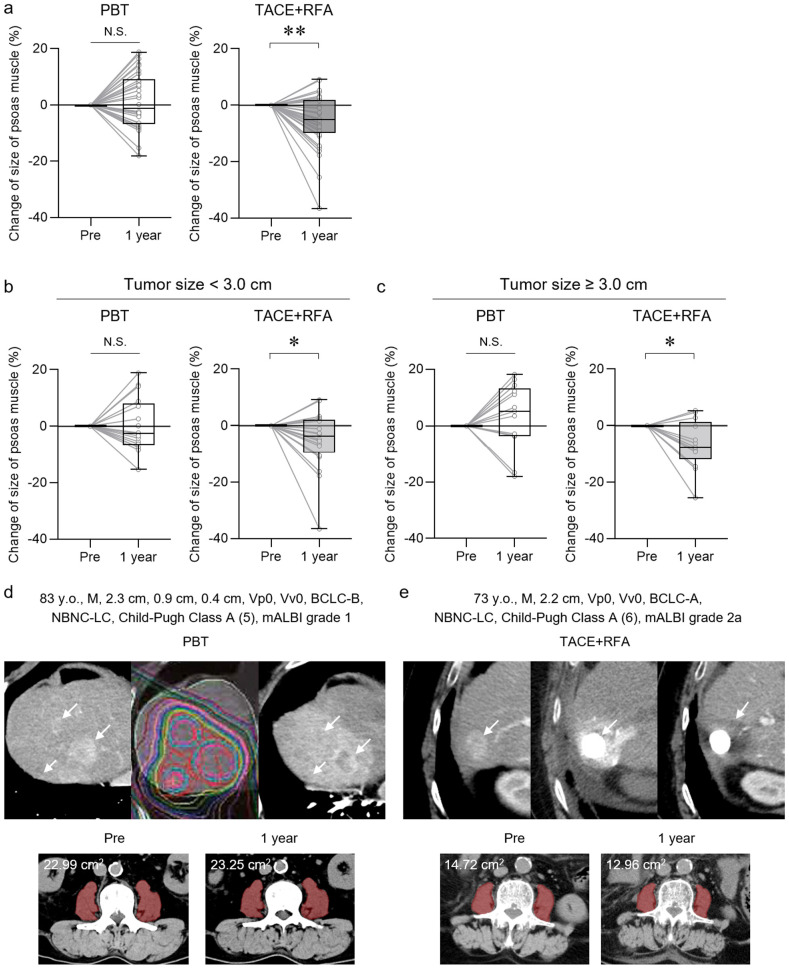
Changes in the size of the psoas muscle after PBT and TACE+RFA treatment in patients with HCC. (**a**) Changes in the size of the psoas muscle after PBT and TACE+RFA treatment for approximately 1 year. Each value represents the box and whisker plot (highest, third quartile, median, first quartile, and lowest value). (**b**,**c**) Changes in the size of the psoas major muscle after approximately 1 year of PBT and TACE+RFA treatment for HCC with a tumor diameter of <3 cm (**b**) and >3 cm (**c**). Each value represents the box and whisker plot (highest, third quartile, median, first quartile, and lowest value). (**d**,**e**) Clinical courses of two representative cases of HCC treated with PBT or TACE+RFA. (**d**) Case of HCC treated with PBT. Imaging findings are shown (white arrows): contrast-enhanced CT in the arterial phase before treatment (left), PBT dose distribution (center), and contrast-enhanced CT in the arterial phase 1 year after PBT (right). Changes in the psoas muscle area at the L3 level following treatment are also demonstrated. (**e**) Case of HCC treated with TACE+RFA. Imaging findings are shown (white arrows): contrast-enhanced CT in the arterial phase before treatment (left), plain CT after TACE (center), and contrast-enhanced CT in the arterial phase after RFA (right). Changes in the psoas muscle area at the L3 level following treatment are also presented. (**a**–**c**) Wilcoxon signed rank test. * *p* < 0.05, ** *p* < 0.01, N.S., not significant. PBT, proton beam therapy; TACE, transarterial chemoembolization; RFA, radiofrequency ablation.

The psoas muscle area at the L3 level of the lumbar spine was measured from CT images acquired before and after treatment with PBT and TACE+RFA. No change in the size of the psoas muscle area after approximately 1 year was observed in the PBT group (mean ± SD [%]; 1.41 ± 9.97) (Figure 3a). In the TACE+RFA group, the size of the psoas muscle area significantly decreased after approximately 1 year (mean ± SD [%]; −5.75 ± 9.60). In patients treated with PBT, the size of the psoas major muscle did not decrease, regardless of whether the tumor size was <3 cm or ≥3 cm (Figure 3b,c). Two representative cases of HCC treated with curative locoregional therapies are shown (Figure 3d,e). The first case was an 83-year-old male with three lesions measuring 2.3 cm, 0.9 cm, and 0.4 cm in diameter, who was treated with PBT at a total dose of 72.6 GyE delivered in 22 fractions of 3.3 GyE each (Figure 3d). The second case was a 73-year-old male with a solitary lesion measuring 2.2 cm in diameter, who was treated with TACE+RFA (Figure 3e). In both cases, the HCC tumors were curatively controlled by the treatments. In the first case treated with PBT, the size of psoas muscle area was maintained after approximately 1 year (+1.1%) (Figure 3d). In contrast, in the second case treated with TACE+RFA, the size of psoas muscle area decreased after approximately 1 year (−12.0%) (Figure 3e). In the TACE+RFA group, the size of the psoas muscle decreased regardless of tumor size (<3 cm or ≥3 cm). These results indicate that, regardless of tumor size, the psoas muscle area does not decrease after PBT in patients with HCC but decreases after TACE+RFA.

### 3.3. Progression of Muscle Atrophy After Treatment and Survival Time

**Figure 4:**
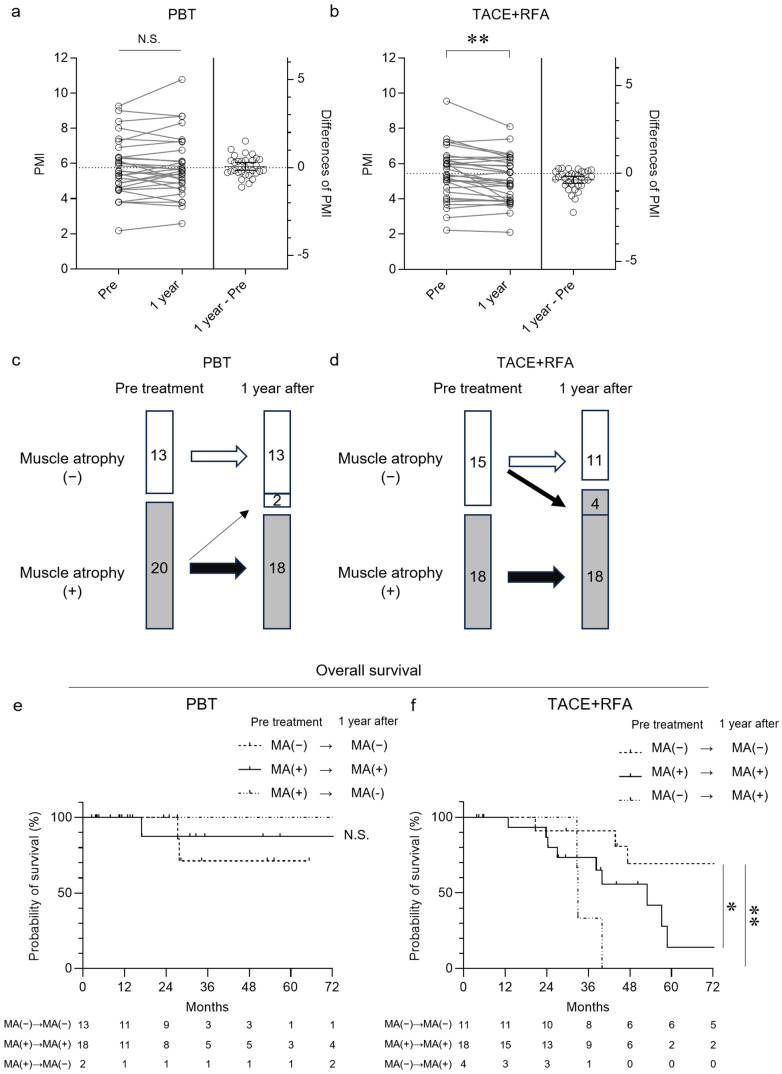
Progression of muscle atrophy after PBT and TACE+RFA treatment and survival time in patients with HCC. (**a**,**b**) Changes in PMI (psoas muscle index) before treatment and approximately 1 year after PBT (**a**) and TACE+RFA treatment (**b**). (**c**,**d**) The condition of muscle atrophy in patients with HCC who underwent PBT (**c**) and TACE+RFA (**d**) treatment before and approximately 1 year after treatment. (**e**,**f**) Kaplan–Meier curves for OS in patients with HCC who received PBT (**e**) and TACE+RFA (**f**) treatment, stratified by muscle atrophy condition before treatment and approximately 1 year later. (**a**,**b**) Wilcoxon signed rank test. (**e**,**f**) Tukey–Kramer post hoc test. * *p* < 0.05, ** *p* < 0.01, N.S., not significant. MA, muscle atrophy; PBT, proton beam therapy; PMI, psoas muscle index; TACE, transarterial chemoembolization; RFA, radiofrequency ablation.

Muscle atrophy (MA) was defined according to sarcopenia diagnostic criteria using psoas muscle size [28]. Changes in PMI before treatment and approximately 1 year after treatment in the PBT group and the TACE+RFA group were analyzed. The results showed that no significant changes in PMI were observed in the PBT group even after treatment, whereas a significant decrease in PMI was confirmed in the TACE+RFA group (Figure 4a,b). In PBT-treated patients, 20 of 33 patients had MA before treatment (Figure 4c). One year after PBT, 2 out of 20 patients with MA [MA(+)] showed improvement, and 13 patients without MA [MA(−)] showed no progression to MA. In the TACE+RFA group, 18 patients with MA showed no improvement in MA after approximately 1 year, and 4 out of 15 patients without MA showed progression to MA (Figure 4d). When comparing the OS for MA before treatment and approximately 1 year later, no significant difference was observed in the PBT group (Figure 4e). Compared with the [MA(−)→MA(−)] group, the prognosis was significantly poorer for the [MA(+)→MA(+)] and [MA(−)→MA(+)] groups in patients treated with TACE+RFA (Figure 4f). These results suggest that PBT does not lead to MA and that MA after approximately 1 year is not associated with a poor prognosis. Meanwhile, TACE+RFA promotes MA, and MA after approximately 1 year is suggested to be associated with a poor prognosis.

### 3.4. Adverse Events

In the PBT group, the incidence of radiation pneumonitis was higher than that in the TACE+RFA group, but all cases were grade 1 (Table 4). The incidence of ALT/AST increase and fever was higher with TACE and RFA than with PBT. In the PBT group, grade 3 or higher adverse events did not occur, but in the TACE+RFA group, grade 3 or 4 increases in AST/ALT were observed. These results indicate that the incidence of hepatotoxicity, general disorders, and grade 3 and 4 adverse events was lower with PBT than with TACE and RFA.

## 4. Discussion

PBT treatment was effective for patients with HCC, resulting in a longer OS than TACE+RFA. Regardless of tumor size, the psoas muscle area did not decrease after PBT but significantly decreased after TACE+RFA. PBT did not cause MA, and MA after approximately 1 year was not associated with prognosis. However, TACE+RFA promoted MA, and MA after approximately 1 year was associated with poor prognosis. The rate of hepatic and systemic adverse events was lower with PBT than with TACE+RFA.

Sarcopenia, a progressive systemic muscle disease characterized by a decrease in muscle mass and muscle strength, is an important complication of chronic liver disease [28]. Methods of measuring body composition include subcutaneous fat thickness evaluation, bioelectrical impedance analysis, dual-energy X-ray absorptiometry, CT, and quantitative MRI [2,30]. Since CT is commonly used to evaluate treatment effects in patients with HCC, it is considered the most suitable method for assessing skeletal muscle [2]. PMI is a more practical method for evaluating muscle mass than SMI, and correlations with SMI have also been reported [31]. In patients with HCC, the annual rate of change in skeletal muscle area is an important factor. The relationship between changes in body composition over time and clinical outcomes has been reported for drug therapies such as sorafenib [32], lenvatinib [2,32], and atezolizumab plus bevacizumab [2]. To our knowledge, this is the first study to evaluate changes in the area of the psoas muscle and survival time after PBT in patients with HCC.

In this study, PBT did not decrease the size of the psoas muscle after treatment. Three possible reasons may explain this. The first is that PBT has low hepatotoxicity. PBT can significantly reduce the dose to non-target liver tissue in patients with unresectable HCC, achieving excellent long-term tumor control with minimal toxicity [11,33]. The effect of PBT in reducing the risk of RILD has been demonstrated to increase with tumor size [34]. Second, PBT has lower toxicity in organs other than the liver. A previous study reported no grade 3 or higher acute or late adverse events in the PBT group, and skin and soft tissue adverse events were manageable [33]. Patients who received PBT tended to have fewer hospital days during treatment than those who received TACE [35]. In this study, all patients completed treatment as outpatients. The third factor is the progression of HCC and cirrhosis, which can lead to secondary sarcopenia [28,32]. PBT ensures a wide margin unaffected by the cooling effect. This prevents the recurrence of satellite lesions near the target lesion, resulting in a lower recurrence rate of non-target lesions than that with RFA [33]. Regarding hepatic reserve function, previous reports indicated no worsening of the ALBI score in the PBT group during the first year after treatment; however, the ALBI score worsened in the TACE+RFA group [13].

In this study, muscle atrophy in the PBT group was not associated with a significant reduction in overall survival. Several factors may contribute to this finding. First, no significant decrease in skeletal muscle mass was observed in the PBT group even one year after treatment, and muscle mass was largely maintained at pre-treatment levels. Previous studies have shown that in patients with HCC, muscle loss during or after treatment—rather than baseline sarcopenia—is a significant prognostic factor for overall survival. For instance, in patients treated with TACE [36,37,38] or molecular targeted agents such as lenvatinib [39] and sorafenib [40], treatment-related muscle wasting has been reported as an independent predictor of poor prognosis. Progressive skeletal muscle loss has been associated with systemic pathophysiological changes such as impaired hepatic glycogen synthesis, hyperammonemia, inflammatory cytokine production, and endocrine alterations [41,42], all of which reflect a decline in overall physiological status. Second, better preservation of liver function after treatment may have contributed to the observed outcome in the PBT group. As we previously reported, PBT offers superior hepatic sparing due to its excellent dose concentration compared to TACE+RFA [13]. Liver functional reserve is a critical determinant of prognosis in patients with HCC, strongly associated with overall survival, recurrence, and the risk of post-hepatectomy liver failure [43]. Therefore, the favorable hepatic function preservation achieved with PBT may have attenuated the negative prognostic impact typically associated with muscle atrophy.

In the present study, TACE+RFA caused a decrease in skeletal muscle mass after treatment. Patients who developed MA had a poor prognosis. This is consistent with previous research showing that a rapid decrease in skeletal muscle mass is associated with a poor prognosis in patients with HCC treated with TACE [37]. Additionally, sarcopenia before TACE is an independent risk factor for poor prognosis, consistent with previous studies [44]. Adverse events, including hepatic dysfunction of grade 3 or higher, fatigue, and fever, occurred in both the TACE and RFA groups. Moreover, additional treatment for recurrence at non-target sites was reported to be a risk factor for decreased skeletal muscle mass [28,32].

This study has several limitations. First, this was a retrospective, non-randomized study conducted at a single institution; therefore, the possibility of selection bias cannot be excluded. In addition, the relatively small sample size may limit the generalizability of the findings. As all patients in this study were treated at institutions located in Fukui, Japan, the generalizability of the findings to more diverse populations may be limited. To address these limitations, we newly applied PSM to adjust for confounding factors such as age, gender, ECOG-PS, etiology, muscle atrophy, AFP, ALBI score, tumor size, number of treated lesions, and vascular invasion. Second, 14.6% of the PBT group and 28.0% of the TACE+RFA group received other treatments for recurrence at target and non-target sites within approximately 1 year of treatment. Consequently, estimating the effect of PBT or TACE+RFA alone on the size of the psoas muscle or the response to treatment was not possible. To clarify the effect of PBT or TACE+RFA on the size of the psoas muscle, hepatic reserve function, and OS, a larger-cohort, multicenter, prospective study should be conducted. Third, we were unable to accurately assess alcohol consumption in some cases, which precluded the specific identification of alcohol-related hepatocellular carcinoma. As such, the etiology was broadly classified as viral (HBV or HCV) or non-viral (NBNC). This may limit the generalizability of our findings, particularly to populations with a high prevalence of alcohol-related liver disease.

## 5. Conclusions

For patients with unresectable HCC not adequately controlled by RFA as monotherapy, PBT may offer prolonged overall survival and better preservation of psoas muscle size. Compared to TACE+RFA, PBT demonstrated lower hepatic and systemic toxicity and more favorable clinical outcomes. These findings suggest that PBT could represent an effective and preferable treatment for unresectable HCC.

## Figures and Tables

**Table 1:** Baseline characteristics of patients in the total and propensity score–matched cohorts treated with proton beam therapy and TACE+RFA.

Characteristics	Total Cohort			PSM Cohort		
	PBT (*n* = 41)	TACE+RFA (*n* = 50)	*p* Value	PBT (*n* = 33)	TACE+RFA (*n* = 33)	*p* Value
Age, years						
Median (IQR)	74 (67–77)	75 (70–81)	0.342 *	75 (70–80)	73 (68–76)	0.228 *
<65	4 (9.8)	3 (6.0)	0.702 ^†^	2 (6.1)	3 (9.1)	>0.999 ^†^
≥65	37 (90.2)	43 (94.0)		31 (93.9)	30 (90.9)	
Gender						
Male	29 (70.7)	34 (68.0)	0.823 ^†^	22 (66.7)	24 (72.7)	0.789 ^†^
Female	12 (29.3)	16 (32.0)		11 (33.3)	9 (27.3)	
ECOG performance status						
0	36 (87.8)	41 (82.0)	0.564 ^†^	28 (84.8)	32 (97.0)	0.197 ^†^
1	5 (12.2)	9 (18.0)		5 (15.2)	1 (3.0)	
BMI, kg/m^2^, median (IQR)	25.1 (22.4–27.3)	23.3 (20.8–25.6)	0.063 *	25.1 (22.4–27.2)	23.0 (20.0–24.9)	0.064 *
Baseline PMI in men, cm^2^/m^2^, median (IQR)	6.15 (5.49–7.09)	5.98 (5.20–6.51)	0.413 *	6.15 (5.43–7.27)	5.98 (5.22–6.40)	0.450 *
Baseline PMI in women, cm^2^/m^2^, median (IQR)	4.52 (3.79–4.99)	4.57 (1.96–3.94)	0.828 *	4.50 (3.79–5.08)	4.05 (3.68–4.62)	0.642 *
Etiology						
HBV	5 (12.2)	2 (4.0)	0.073 ^‡^	5 (15.2)	2 (6.1)	0.348 ^‡^
HCV	9 (22.0)	21 (42.0)		8 (24.2)	12 (36.4)	
NBNC	27 (65.8)	27 (54.0)		20 (60.6)	19 (57.6)	
Neutrophils, ×103/m^3^, median (IQR)	2548 (1848–3203)	2763 (1979–4071)	0.243 *	2459 (1825–3203)	2582 (1544–4062)	0.738 *
Lymphocytes, ×103/m^3^, median (IQR)	1231 (918–1760)	1267 (879–1736)	0.945 *	1158 (900–1688)	1243 (875–1742)	0.709 *
NLR, median (IQR)	1.92 (1.45–2.84)	2.34 (1.63–3.37)	0.197 *	2.19 (1.58–2.92)	2.03 (1.53–2.94)	0.801 *
Choline-esterase, U/L, median (IQR)	235 (158–282)	188 (155–243)	0.132 *	223 (149–282)	186 (154–243)	0.306 *
Total cholesterol, mg/dL, median (IQR)	174 (151–195)	155 (135–179)	0.055 *	174 (151–197)	155 (136–172)	0.061 *
LDL-cholesterol, mg/dL, median (IQR)	89 (72–106)	83 (71–101)	0.297 *	89 (72–105)	81 (72–100)	0.245 *
CRP, mg/dL, median (IQR)	0.13 (0.07–0.36)	0.14 (0.05–0.32)	0.618 *	0.13 (0.08–0.24)	0.11 (0.05–0.24)	0.405 *
Hemoglobin A1c, %, median (IQR)	5.9 (5.3–6.6)	6.0 (5.5–6.8)	0.386 *	6.1 (5.2–6.6)	6.1 (5.6–6.8)	0.489 *
Hyaluronic acid, ng/mL, median (IQR)	159.5 (88.2–263.0)	192.0 (125.0–256.0)	0.380 *	174 (110–275)	191 (125–221)	0.815 *
Type IV collagen 7S, ng/mL, median (IQR)	2.10 (1.26–3.81)	2.56 (1.51–5.20)	0.558 *	6.40 (5.60–9.10)	2.62 (1.74–4.86)	0.898 *
M2BPGi, C.O.I, median (IQR)	6.20 (5.35–7.80)	6.55 (5.10–8.98)	0.825 *	2.29 (1.28–5.67)	6.6 (5.2–9.0)	0.947 *
AFP, ng/Ml ^#^	4.2 (1.1–13099)	8.1 (0.5–1922.9)	0.156 *	3.9 (1.1–457.7)	7.9 (1.4–149.6)	0.257 *
<13.4	30 (73.2)	33 (66.0)	0.501 ^†^	24 (72.7)	25 (75.8)	>0.999 ^†^
≥13.4	11 (26.8)	17 (34.0)		9 (27.3)	8 (24.2)	
DCP, mAU/mL ^#^	36 (8–22694)	38 (7–50929)	0.926 *	29 (8–535)	43 (7–50929)	0.227 *
<100	29 (70.7)	36 (72.0)	>0.999 ^†^	25 (80.6)	23 (69.7)	0.392 ^†^
≥100	12 (29.3)	14 (28.0)		6 (19.4)	10 (30.3)	
Child-Pugh score						
5	19 (46.3)	30 (60.0)	0.212 ^†^	16 (48.5)	22 (66.7)	0.213 ^†^
≥6	22 (53.7)	20 (40.0)		17 (51.5)	11 (33.3)	
mALBI grade						
1, 2a	27 (65.9)	35 (70.0)	0.822 ^†^	20 (60.6)	23 (69.7)	0.606 ^†^
2b, 3	14 (34.1)	15 (30.0)		13 (39.4)	10 (30.3)	
Tumor size, cm ^#^	2.6 (1.2–9.3)	2.6 (1.3–7.0)	0.957 *	2.3 (1.2–7.1)	2.6 (1.3–7.0)	0.603 *
<3	23 (56.1)	34 (68.0)	0.281 ^†^	20 (60.6)	21 (63.6)	>0.999 ^†^
≥3	18 (43.9)	16 (32.0)		13 (39.4)	12 (36.4)	
Number of treated lesion(s)						
1	35 (85.4)	37 (74.0)	0.335 ^‡^	27 (81.8)	26 (78.8)	0.582 ^‡^
2	6 (14.6)	12 (24.0)		6 (18.2)	7 (21.2)	
3	0 (0.0)	1 (2.0)		0 (0.0)	0 (0.0)	
Vascular invasion						
absent	36 (87.8)	49 (98.0)	0.087 ^†^	33 (100.0)	32 (97.0)	>0.999 ^†^
present	5 (12.2)	1 (2.0)		0 (0.0)	1 (3.0)	
BCLC stage						
0	6 (14.6)	4 (12.0)	0.579 ^†^	5 (15.2)	4 (12.1)	0.792 ^†^
A	30 (73.2)	36 (72.0)		23 (69.7)	22 (66.7)	
B	5 (12.2)	8 (16.0)		5 (15.2)	7 (21.2)	
CT follow-up periods, months, median (IQR)	11.9 (8.3–14.6)	12.1 (10.9–14.3)	0.528 *	11.9 (10.0–14.9)	12.1 (11.2–15.1)	0.581 *
Follow-up time, months, median (IQR)	26.1 (11.1–52.4)	38.2 (20.1–49.7)	0.143 *	24.9 (10.6–35.3)	37.5 (20.0–49.9)	0.110 *

AFP, alpha fetoprotein; BCLC stage, Barcelona Clinic Liver Cancer stage; BMI, body mass index; CRP, C-reactive protein; DCP, des-gamma-carboxy prothrombin; ECOG, Eastern Cooperative Oncology Group; IQR, interquartile range; LDL, low-density lipoprotein; M2BPGi, mac-2 binding protein glycosylation isomer; mALBI, modified albumin–bilirubin; NBNC, nonB-nonC; NLR, neutrophil-to-lymphocyte ratio; PBT, proton beam therapy; PSM, propensity score matching; RFA, radiofrequency ablation; TACE, transarterial chemoembolization; PMI, psoas muscle index. ^#^, Continuous variables are presented as median (range). *, Mann–Whitney U test. ^†^, Fisher’s exact test. ^‡^, Chi-square test.

**Table 2:** Post-treatment management of target lesions in the total and propensity score–matched cohorts treated with proton beam therapy and TACE+RFA.

Characteristics	Total Cohort			PSM Cohort		
	PBT (*n* = 41)	TACE+RFA (*n* = 50)	*p* Value	PBT (*n* = 33)	TACE+RFA (*n* = 33)	*p* Value
Post treatment to target lesion(s)						
No	37 (90.2)	37 (74.0)	0.061 ^†^	30 (90.9)	20 (60.6)	0.061 ^†^
Yes	4 (9.8)	13 (26.0)		3 (9.1)	10 (30.3)	
RFA	0 (0.0)	1 (2.0)		0 (0.0)	1 (3.0)	
RFA, TACE	0 (0.0)	3 (6.0)		0 (0.0)	2 (6.1)	
RFA (PEIT), TACE, TKI, ICI	0 (0.0)	2 (4.0)		0 (0.0)	1 (3.0)	
TACE	2 (4.8)	3 (6.0)		2 (6.1)	2 (6.1)	
TACE, TKI, ICI	1 (2.4)	1 (2.0)		0 (0.0)	1 (3.0)	
TACE, ICI	0 (0.0)	1 (2.0)		0 (0.0)	1 (3.0)	
TKI	0 (0.0)	1 (2.0)		0 (0.0)	1 (3.0)	
TKI, HAIC	1 (2.4)	1 (2.0)		1 (3.0)	1 (3.0)	

HAIC, hepatic arterial infusion chemotherapy; ICI, immune checkpoint inhibitor (atezolizumab plus bevacizumab or tremelimumab and durvalumab); PBT, proton beam therapy; PEIT, percutaneous ethanol injection therapy; PSM, propensity score matching; RFA, radiofrequency ablation; TACE, transarterial chemoembolization; TKI, tyrosine kinase inhibitor. ^†^, Fisher’s exact test.

**Table 3:** Multivariate analysis of clinical factors associated with survival in patients with hepatocellular carcinoma.

		Multivariate Analysis		
Variables	Patients (*n* = 66)	Odds Ratio	95% CI	*p* Value
Age, y, (≤70/>70)	17/49	0.122	−1.326–0.906	0.727
Gender, (Male/Female)	46/20	1.460	−0.493–2.041	0.227
ECOG-PS, (0/1)	60/6	0.132	−3.481–1.641	0.716
Etiology, (HBV, HCV/NBNC)	27/39	2.423	−0.240–2.184	0.120
Muscle atrophy (atrophy−/atrophy+)	28/38	3.312	−0.084–2.483	0.069
AFP, (<13.4/≥13.4)	49/17	0.792	−0.883–2.062	0.374
mALBI grade, (1, 2a/2b,3)	44/22	4.545	0.119–2.825	0.033
Tumor size, mm, (<30/≥30)	41/25	0.089	−1.296–0.959	0.766
Number of treated lesion(s) (1/2)	53/13	1.196	−0.677–2.305	0.274
Vascular invasion (absent/present)	65/1	0.891	−1.926–4.478	0.345
Treatment (PBT/TACE+RFA)	33/33	6.297	0.3017–3.040	0.012

AFP, alpha fetoprotein; ECOG, Eastern Cooperative Oncology Group; mALBI, modified albumin–bilirubin; NBNC, nonB-nonC; PBT, proton beam therapy; PSM, propensity score matching; RFA, radiofrequency ablation; TACE, transarterial chemoembolization; PMI, psoas muscle index.

**Table 4:** Adverse events of propensity score-matched cohorts after proton beam therapy and TACE+RFA.

					TACE+RFA (*n* = 33), *n* (%)									
	PBT (*n* = 33), *n* (%)				TACE (*n* = 33), *n* (%)				PBT vs. TACE	RFA (*n* = 33), *n* (%)				PBT vs. RFA
CTCAE Grade	Grade 1	Grade 2	Grade 3	Grade 4	Grade 1	Grade 2	Grade 3	Grade 4	*p* Value	Grade 1	Grade 2	Grade 3	Grade 4	*p* Value
ALT/AST increase	0 (0.0)	0 (0.0)	0 (0.0)	0 (0.0)	13 (39.4)	6 (18.2)	6 (18.2)	1 (3.0)	<0.001 ^‡^	16 (48.5)	10 (30.3)	6 (18.2)	0 (0.0)	<0.001 ^‡^
Albumin decrease	2 (6.1)	0 (0.0)	0 (0.0)	0 (0.0)	6 (18.2)	0 (0.0)	0 (0.0)	0 (0.0)	0.258 ^†^	5 (15.2)	0 (0.0)	0 (0.0)	0 (0.0)	0.427 ^†^
Bilirubin increase	7 (21.2)	0 (0.0)	0 (0.0)	0 (0.0)	12 (36.4)	2 (6.1)	0 (0.0)	0 (0.0)	0.111 ^‡^	9 (27.3)	3 (9.1)	0 (0.0)	0 (0.0)	0.201 ^‡^
Fever	0 (0.0)	0 (0.0)	0 (0.0)	0 (0.0)	9 (27.3)	8 (24.2)	0 (0.0)	0 (0.0)	<0.001 ^‡^	14 (42.4)	4 (12.1)	0 (0.0)	0 (0.0)	<0.001 ^‡^
Pain	0 (0.0)	0 (0.0)	0 (0.0)	0 (0.0)	2 (6.1)	1 (3.0)	0 (0.0)	0 (0.0)	0.208 ^‡^	2 (6.1)	0 (0.0)	0 (0.0)	0 (0.0)	0.492 ^†^
Nausea	0 (0.0)	0 (0.0)	0 (0.0)	0 (0.0)	6 (18.2)	5 (15.2)	0 (0.0)	0 (0.0)	<0.001 ^‡^	2 (6.1)	3 (9.1)	0 (0.0)	0 (0.0)	0.067 ^‡^
Dermatitis	1 (3.0)	0 (0.0)	0 (0.0)	0 (0.0)	0 (0.0)	0 (0.0)	0 (0.0)	0 (0.0)	>0.999 ^†^	0 (0.0)	0 (0.0)	0 (0.0)	0 (0.0)	>0.999 ^†^
Radiation pneumonitis	10 (30.3)	0 (0.0)	0 (0.0)	0 (0.0)	0 (0.0)	0 (0.0)	0 (0.0)	0 (0.0)	<0.001 ^†^	0 (0.0)	0 (0.0)	0 (0.0)	0 (0.0)	<0.001 ^†^
Pleural effusion	2 (6.1)	0 (0.0)	0 (0.0)	0 (0.0)	0 (0.0)	0 (0.0)	0 (0.0)	0 (0.0)	0.492 ^†^	0 (0.0)	0 (0.0)	0 (0.0)	0 (0.0)	0.492 ^†^
Ascites	0 (0.0)	0 (0.0)	0 (0.0)	0 (0.0)	2 (6.1)	0 (0.0)	0 (0.0)	0 (0.0)	0.492 ^†^	5 (15.2)	0 (0.0)	0 (0.0)	0 (0.0)	0.053 ^†^
No. of patients with Grade 3 and 4 AEs	0 (0.0)				7 (21.2)				0.011 ^†^	6 (18.2)				0.024 ^†^

AE, adverse event; ALT, alanine aminotransferase; AST, aspartate aminotransferase; CTCAE, common terminology criteria for adverse events; PBT, proton beam radiotherapy; RFA, radiofrequency ablation. ^†^, Fisher’s exact test. ^‡^, Chi-square test.

## Data Availability

The data of the current study are available from the corresponding author upon reasonable request. The data are not publicly available due to privacy and ethical reasons.

## Supplementary Materials

The following supporting information can be downloaded at: https://www.mdpi.com/article/10.3390/cancers17172849/s1, Figure S1: Study flowchart showing inclusion and exclusion criteria and propensity score matching.

## References

[1] Siegel R.L., Miller K.D., Wagle N.S., Jemal A. (2023). Cancer statistics, 2023. CA Cancer J. Clin..

[2] Imai K., Takai K., Unome S., Miwa T., Hanai T., Suetsugu A., Shimizu M. (2024). Lenvatinib Exacerbates the Decrease in Skeletal Muscle Mass in Patients with Hepatocellular Carcinoma, Whereas Atezolizumab Plus Bevacizumab Does Not. Cancers.

[3] Lai J.C., Tandon P., Bernal W., Tapper E.B., Ekong U., Dasarathy S., Carey E.J. (2021). Malnutrition, Frailty, and Sarcopenia in Patients With Cirrhosis: 2021 Practice Guidance by the American Association for the Study of Liver Diseases. Hepatology.

[4] Imai K., Takai K., Hanai T., Ideta T., Miyazaki T., Kochi T., Suetsugu A., Shiraki M., Shimizu M. (2015). Skeletal muscle depletion predicts the prognosis of patients with hepatocellular carcinoma treated with sorafenib. Int. J. Mol. Sci..

[5] Iritani S., Imai K., Takai K., Hanai T., Ideta T., Miyazaki T., Suetsugu A., Shiraki M., Shimizu M., Moriwaki H. (2015). Skeletal muscle depletion is an independent prognostic factor for hepatocellular carcinoma. J. Gastroenterol..

[6] Tantai X., Liu Y., Yeo Y.H., Praktiknjo M., Mauro E., Hamaguchi Y., Engelmann C., Zhang P., Jeong J.Y., van Vugt J.L.A. (2022). Effect of sarcopenia on survival in patients with cirrhosis: A meta-analysis. J. Hepatol..

[7] Hanai T., Shiraki M., Ohnishi S., Miyazaki T., Ideta T., Kochi T., Imai K., Suetsugu A., Takai K., Moriwaki H. (2016). Rapid skeletal muscle wasting predicts worse survival in patients with liver cirrhosis. Hepatol. Res..

[8] Kim T.H., Jung Y.K., Yim H.J., Baik J.W., Yim S.Y., Lee Y.-S., Seo Y.S., Kim J.H., Yeon J.E., Byun K.S. (2022). Impacts of muscle mass dynamics on prognosis of outpatients with cirrhosis. Clin. Mol. Hepatol..

[9] Bush D.A., Kayali Z., Grove R., Slater J.D. (2011). The safety and efficacy of high-dose proton beam radiotherapy for hepatocellular carcinoma: A phase 2 prospective trial. Cancer.

[10] Hong T.S., Wo J.Y., Yeap B.Y., Ben-Josef E., McDonnell E.I., Blaszkowsky L.S., Kwak E.L., Allen J.N., Clark J.W., Goyal L. (2016). Multi-Institutional Phase II Study of High-Dose Hypofractionated Proton Beam Therapy in Patients With Localized, Unresectable Hepatocellular Carcinoma and Intrahepatic Cholangiocarcinoma. J. Clin. Oncol..

[11] Chuong M., Kaiser A., Molitoris J., Romero A.M., Apisarnthanarax S. (2020). Proton beam therapy for liver cancers. J. Gastrointest. Oncol..

[12] Toramatsu C., Katoh N., Shimizu S., Nihongi H., Matsuura T., Takao S., Miyamoto N., Suzuki R., Sutherland K., Kinoshita R. (2013). What is the appropriate size criterion for proton radiotherapy for hepatocellular carcinoma? A dosimetric comparison of spot-scanning proton therapy versus intensity-modulated radiation therapy. Radiat. Oncol..

[13] Nosaka T., Matsuda H., Sugata R., Akazawa Y., Takahashi K., Naito T., Ohtani M., Kinoshita K., Tsujikawa T., Sato Y. (2023). Longer Survival and Preserved Liver Function after Proton Beam Therapy for Patients with Unresectable Hepatocellular Carcinoma. Curr. Oncol..

[14] Chen L., Sun J., Yang X. (2016). Radiofrequency ablation-combined multimodel therapies for hepatocellular carcinoma: Current status. Cancer Lett..

[15] Lee S., Kang T.W., Cha D.I., Song K.D., Lee M.W., Rhim H., Lim H.K., Sinn D.H., Kim J.M., Kim K. (2018). Radiofrequency ablation vs. surgery for perivascular hepatocellular carcinoma: Propensity score analyses of long-term outcomes. J. Hepatol..

[16] Cao S., Zou Y., Lyu T., Fan Z., Guan H., Song L., Tong X., Wang J. (2022). Long-term outcomes of combined transarterial chemoembolization and radiofrequency ablation versus RFA monotherapy for single hepatocellular carcinoma ≤3 cm: Emphasis on local tumor progression. Int. J. Hyperthermia.

[17] Hatzidakis A., Müller L., Krokidis M., Kloeckner R. (2022). Local and Regional Therapies for Hepatocellular Carcinoma and Future Combinations. Cancers.

[18] Ren Y., Cao Y., Ma H., Kan X., Zhou C., Liu J., Shi Q., Feng G., Xiong B., Zheng C. (2019). Improved clinical outcome using transarterial chemoembolization combined with radiofrequency ablation for patients in Barcelona clinic liver cancer stage A or B hepatocellular carcinoma regardless of tumor size: Results of a single-center retrospective case control study. BMC Cancer.

[19] Yang Y., Yu H., Qi L., Liu C., Feng Y., Qi J., Li J., Zhu Q. (2022). Combined radiofrequency ablation or microwave ablation with transarterial chemoembolization can increase efficiency in intermediate-stage hepatocellular carcinoma without more complication: A systematic review and meta-analysis. Int. J. Hyperthermia.

[20] Hiraoka A., Kumada T., Kudo M., Hirooka M., Koizumi Y., Hiasa Y., Tajiri K., Toyoda H., Tada T., Ochi H. (2017). Hepatic Function during Repeated TACE Procedures and Prognosis after Introducing Sorafenib in Patients with Unresectable Hepatocellular Carcinoma: Multicenter Analysis. Digestive. Diseases..

[21] Jing C., Li J., Yuan C., Hu C., Ma L., Zheng J., Zhang Y. (2024). Therapeutic analysis of 632 cases treated by transcatheter arterial chemoembolization combined with ablation in hepatocellular carcinoma: A retrospective study. Eur. J. Radiol..

[22] Shimose S., Tanaka M., Iwamoto H., Niizeki T., Shirono T., Aino H., Noda Y., Kamachi N., Okamura S., Nakano M. (2019). Prognostic impact of transcatheter arterial chemoembolization (TACE) combined with radiofrequency ablation in patients with unresectable hepatocellular carcinoma: Comparison with TACE alone using decision-tree analysis after propensity score matching. Hepatol. Res..

[23] Singal A.G., Llovet J.M., Yarchoan M., Mehta N., Heimbach J.K., Dawson L.A., Jou J.H., Kulik L.M., Agopian V.G., Marrero J.A. (2023). AASLD Practice Guidance on prevention, diagnosis, and treatment of hepatocellular carcinoma. Hepatology.

[24] Johnson P.J., Berhane S., Kagebayashi C., Satomura S., Teng M., Reeves H.L., O’Beirne J., Fox R., Skowronska A., Palmer D. (2015). Assessment of liver function in patients with hepatocellular carcinoma: A new evidence-based approach-the ALBI grade. J. Clin. Oncol..

[25] Hiraoka A., Kumada T., Tsuji K., Takaguchi K., Itobayashi E., Kariyama K., Ochi H., Tajiri K., Hirooka M., Shimada N. (2019). Validation of Modified ALBI Grade for More Detailed Assessment of Hepatic Function in Hepatocellular Carcinoma Patients: A Multicenter Analysis. Liver Cancer.

[26] Maeda Y., Kobashi K., Sato Y., Tamamura H., Yamamoto K., Matsushita K., Sasaki M., Tatebe H., Asahi T., Matsumoto S. (2023). Effectiveness of CT-image guidance in proton therapy for liver cancer and the importance of daily dose monitoring for tumors and organs at risk. Med. Phys..

[27] Komatsu S., Fukumoto T., Demizu Y., Miyawaki D., Terashima K., Sasaki R., Hori Y., Hishikawa Y., Ku Y., Murakami M. (2011). Clinical results and risk factors of proton and carbon ion therapy for hepatocellular carcinoma. Cancer.

[28] Nishikawa H., Shiraki M., Hiramatsu A., Moriya K., Hino K., Nishiguchi S. (2016). Japan Society of Hepatology guidelines for sarcopenia in liver disease (1st edition): Recommendation from the working group for creation of sarcopenia assessment criteria. Hepatol. Res..

[29] Kanda Y. (2013). Investigation of the freely available easy-to-use software ‘EZR’ for medical statistics. Bone Marrow Transplant..

[30] Barone M., Losurdo G., Iannone A., Leandro G., Di Leo A., Trerotoli P. (2022). Assessment of body composition: Intrinsic methodological limitations and statistical pitfalls. Nutrition.

[31] Hiraoka A., Aibiki T., Okudaira T., Toshimori A., Kawamura T., Nakahara H., Suga Y., Azemoto N., Miyata H., Miyamoto Y. (2015). Muscle atrophy as pre-sarcopenia in Japanese patients with chronic liver disease: Computed tomography is useful for evaluation. J. Gastroenterol..

[32] Imai K., Takai K., Unome S., Miwa T., Hanai T., Suetsugu A., Shimizu M. (2023). Lenvatinib or Sorafenib Treatment Causing a Decrease in Skeletal Muscle Mass, an Independent Prognostic Factor in Hepatocellular Carcinoma: A Survival Analysis Using Time-Varying Covariates. Cancers.

[33] Sekino Y., Tateishi R., Fukumitsu N., Okumura T., Maruo K., Iizumi T., Numajiri H., Mizumoto M., Minami T., Nakagomi R. (2023). Proton Beam Therapy versus Radiofrequency Ablation for Patients with Treatment-Naïve Single Hepatocellular Carcinoma: A Propensity Score Analysis. Liver Cancer.

[34] Lee S.U., Kim T.H. (2023). Current evidence and the potential role of proton beam therapy for hepatocellular carcinoma. Clin. Mol. Hepatol..

[35] Bush D.A., Volk M., Smith J.C., Reeves M.E., Sanghvi S., Slater J.D., deVera M. (2023). Proton beam radiotherapy versus transarterial chemoembolization for hepatocellular carcinoma: Results of a randomized clinical trial. Cancer.

[36] Fujita M., Takahashi A., Hayashi M., Okai K., Abe K., Ohira H. (2019). Skeletal muscle volume loss during transarterial chemoembolization predicts poor prognosis in patients with hepatocellular carcinoma. Hepatol. Res..

[37] Kobayashi T., Kawai H., Nakano O., Abe S., Kamimura H., Sakamaki A., Kamimura K., Tsuchiya A., Takamura M., Yamagiwa S. (2018). Rapidly declining skeletal muscle mass predicts poor prognosis of hepatocellular carcinoma treated with transcatheter intra-arterial therapies. BMC Cancer.

[38] Yang S., Zhang Z., Su T., Yu J., Cao S., Wang H., Jin L. (2022). CT-based skeletal muscle loss for predicting poor survival in patients with hepatocellular carcinoma experiencing curative hepatectomy plus adjuvant transarterial chemoembolization: A preliminary retrospective study. Eur. J. Med. Res..

[39] Fujita M., Abe K., Kuroda H., Oikawa T., Ninomiya M., Masamune A., Okumoto K., Katsumi T., Sato W., Iijima K. (2022). Influence of skeletal muscle volume loss during lenvatinib treatment on prognosis in unresectable hepatocellular carcinoma: A multicenter study in Tohoku, Japan. Sci. Rep..

[40] Yamashima M., Miyaaki H., Honda T., Shibata H., Miuma S., Taura N., Nakao K. (2017). Significance of psoas muscle thickness as an indicator of muscle atrophy in patients with hepatocellular carcinoma treated with sorafenib. Mol. Clin. Oncol..

[41] Dasarathy S., Merli M. (2016). Sarcopenia from mechanism to diagnosis and treatment in liver disease. J. Hepatol..

[42] Ebadi M., Bhanji R.A., Mazurak V.C., Montano-Loza A.J. (2019). Sarcopenia in cirrhosis: From pathogenesis to interventions. J. Gastroenterol..

[43] Demirtas C.O., D’Alessio A., Rimassa L., Sharma R., Pinato D.J. (2021). ALBI grade: Evidence for an improved model for liver functional estimation in patients with hepatocellular carcinoma. JHEP Rep..

[44] Loosen S.H., Schulze-Hagen M., Bruners P., Tacke F., Trautwein C., Kuhl C., Luedde T., Roderburg C. (2019). Sarcopenia Is a Negative Prognostic Factor in Patients Undergoing Transarterial Chemoembolization (TACE) for Hepatic Malignancies. Cancers.

