# Supplementary material for: Proton Beam Therapy Provides Longer Survival and Preserves Muscle Mass in Hepatocellular Carcinoma Compared to TACE+RFA
Source: Cancers (Basel). 2025 Aug 30;17(17):2849. doi: 10.3390/cancers17172849 (PMC12427364; doi:10.3390/cancers17172849)
Supplement: Supplementary file 1 [file cancers-17-02849-s001.zip › cancers-3789800-supplementary.pdf]

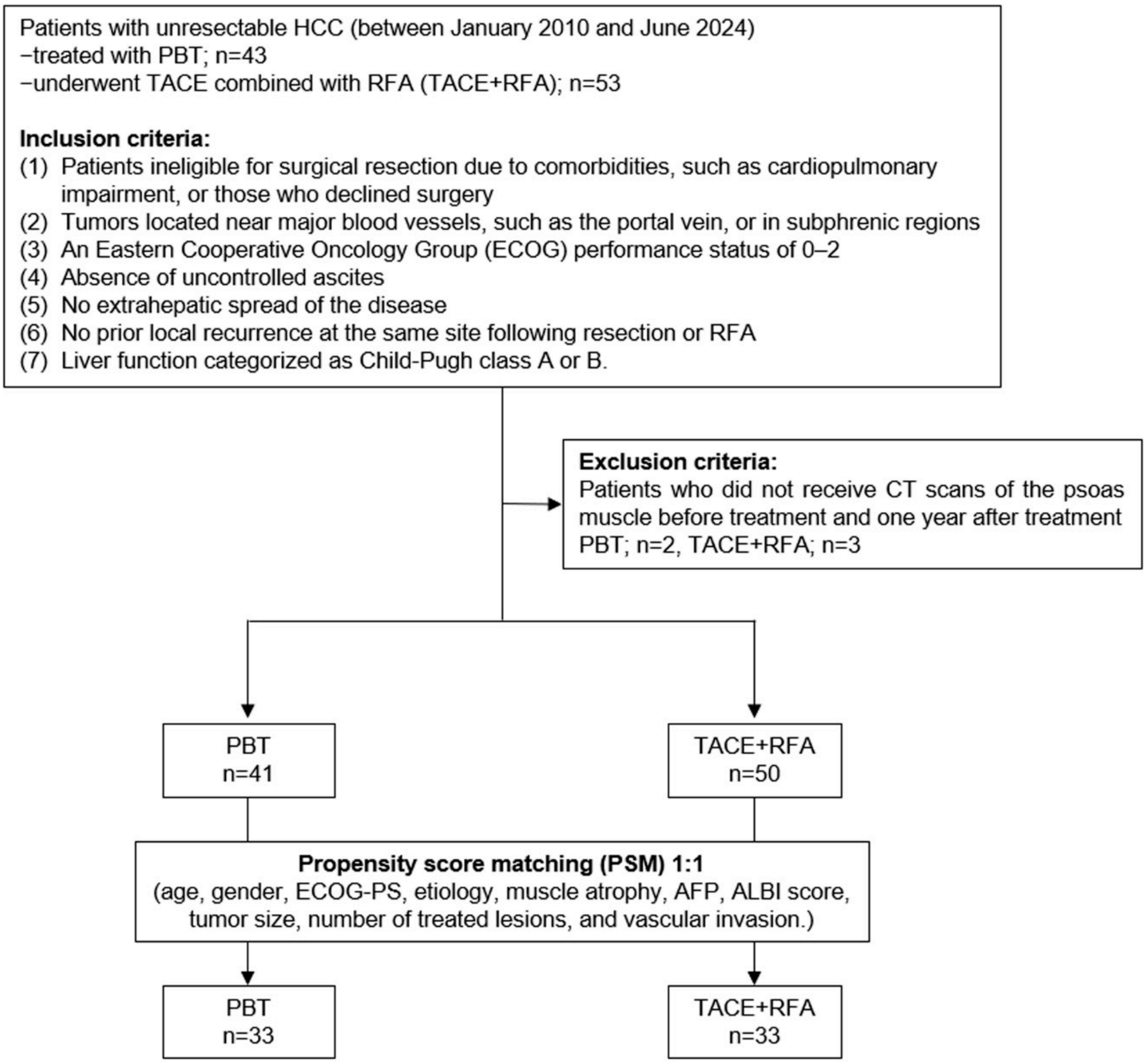

Figure S1. Study flowchart showing inclusion and exclusion criteria and propensity score matching.
